# BloodProST: prediction of blood-secretory proteins through self-training

**DOI:** 10.1093/bib/bbaf385

**Published:** 2025-08-01

**Authors:** Xuechen Mu, Long Xu, Zhenyu Huang, Jing Yan, Bocheng Shi, Yishi Wang, Binyue Liu, Kai Zhang, Ying Xu

**Affiliations:** Key University Laboratory of Metabolism and Health of Guangdong, Southern University of Science and Technology, Shenzhen 518055, China; SUSTech Homeostatic Medicine Institute, School of Medicine, Southern University of Science and Technology, Shenzhen 518055, China; Department of Human Cell Biology and Genetics, SUSTech Homeostatic Medicine Institute, School of Medicine, Southern University of Science and Technology, Shenzhen 518055, Guangdong, China; School of Mathematics, Jilin University, Changchun 130012, Jilin, China; Key University Laboratory of Metabolism and Health of Guangdong, Southern University of Science and Technology, Shenzhen 518055, China; SUSTech Homeostatic Medicine Institute, School of Medicine, Southern University of Science and Technology, Shenzhen 518055, China; Department of Human Cell Biology and Genetics, SUSTech Homeostatic Medicine Institute, School of Medicine, Southern University of Science and Technology, Shenzhen 518055, Guangdong, China; Key University Laboratory of Metabolism and Health of Guangdong, Southern University of Science and Technology, Shenzhen 518055, China; SUSTech Homeostatic Medicine Institute, School of Medicine, Southern University of Science and Technology, Shenzhen 518055, China; Department of Human Cell Biology and Genetics, SUSTech Homeostatic Medicine Institute, School of Medicine, Southern University of Science and Technology, Shenzhen 518055, Guangdong, China; College of Computer Science and Technology, Jilin University, Changchun 130012, Jilin, China; Key University Laboratory of Metabolism and Health of Guangdong, Southern University of Science and Technology, Shenzhen 518055, China; SUSTech Homeostatic Medicine Institute, School of Medicine, Southern University of Science and Technology, Shenzhen 518055, China; Department of Human Cell Biology and Genetics, SUSTech Homeostatic Medicine Institute, School of Medicine, Southern University of Science and Technology, Shenzhen 518055, Guangdong, China; College of Computer Science and Technology, Jilin University, Changchun 130012, Jilin, China; Key University Laboratory of Metabolism and Health of Guangdong, Southern University of Science and Technology, Shenzhen 518055, China; SUSTech Homeostatic Medicine Institute, School of Medicine, Southern University of Science and Technology, Shenzhen 518055, China; Department of Human Cell Biology and Genetics, SUSTech Homeostatic Medicine Institute, School of Medicine, Southern University of Science and Technology, Shenzhen 518055, Guangdong, China; Key University Laboratory of Metabolism and Health of Guangdong, Southern University of Science and Technology, Shenzhen 518055, China; SUSTech Homeostatic Medicine Institute, School of Medicine, Southern University of Science and Technology, Shenzhen 518055, China; Department of Human Cell Biology and Genetics, SUSTech Homeostatic Medicine Institute, School of Medicine, Southern University of Science and Technology, Shenzhen 518055, Guangdong, China; Key University Laboratory of Metabolism and Health of Guangdong, Southern University of Science and Technology, Shenzhen 518055, China; SUSTech Homeostatic Medicine Institute, School of Medicine, Southern University of Science and Technology, Shenzhen 518055, China; Department of Human Cell Biology and Genetics, SUSTech Homeostatic Medicine Institute, School of Medicine, Southern University of Science and Technology, Shenzhen 518055, Guangdong, China; School of Mathematics, Jilin University, Changchun 130012, Jilin, China; Key University Laboratory of Metabolism and Health of Guangdong, Southern University of Science and Technology, Shenzhen 518055, China; SUSTech Homeostatic Medicine Institute, School of Medicine, Southern University of Science and Technology, Shenzhen 518055, China; Department of Human Cell Biology and Genetics, SUSTech Homeostatic Medicine Institute, School of Medicine, Southern University of Science and Technology, Shenzhen 518055, Guangdong, China

**Keywords:** blood secretory proteins, disease diagnostic markers, unsupervised feature selection, machine learning, self-training

## Abstract

Accurate identification of proteins secreted into the bloodstream is essential for discovering diagnostic biomarkers and therapeutic targets. A significant challenge is the scarcity of experimentally validated blood-secretory proteins, limiting labeled datasets required for robust model training. To address this issue, we propose BloodProST, a novel machine-learning framework leveraging a self-training strategy to reliably predict blood-secretory proteins. BloodProST iteratively expands the labeled dataset by generating high-confidence pseudo-labels from a large pool of unlabeled protein sequences, thereby progressively enhancing model predictions without continuous manual annotation. At its core, BloodProST incorporates an unsupervised feature selection module based on differential evolution, optimizing the Silhouette score to identify the most discriminative physicochemical and sequence-derived features. Additionally, BloodProST employs a dual-pathway convolutional neural network and long short-term memory (CNN)-(LSTM) architecture: a CNN-based pathway captures local information from pre-constructed features, whereas an LSTM-based pathway extracts high-level sequential dependencies directly from protein sequences. Furthermore, domain-specific biological priors, such as the expected proportion of secretory proteins, are integrated into the model’s loss function to guide training toward biologically plausible predictions. Extensive evaluation demonstrates that BloodProST significantly outperforms 14 state-of-the-art models across multiple metrics, achieving superior predictive accuracy, robustness, and interpretability. Validation analyses confirm the biological relevance of predictions through secretion-related markers (e.g. signal peptides and transmembrane regions) and demonstrate effective generalization to other biofluids, such as urine. Collectively, these results illustrate BloodProST’s potential as a versatile computational tool for secretion prediction and biomarker discovery across diverse biological fluids.

## Introduction

The human proteome comprises a vast array of proteins, each playing a crucial role in physiological processes and disease progression [1–4]. A significant subset of these proteins can be secreted into the bloodstream, making them highly valuable for clinical diagnostics, including blood-brain barrier studies [5], therapeutic applications, and biomarker development for various diseases [6]. Consequently, accurate prediction of which cellular proteins can be secreted into the bloodstream is of immense importance for biomarker discovery.

Numerous experimental techniques have been developed to determine whether a protein can be secreted into the bloodstream. Technologies such as click chemistry with non-canonical amino acids enable the precise detection of proteins by attaching fluorophores to targeted proteins, allowing for *in vivo* tracking [7]. More general techniques include the Enzyme-Linked Immunosorbent Assay (ELISA), which uses antibodies to detect and quantify specific proteins in blood samples [8], and Western blotting, which can detect which proteins are present in blood samples [9]. Mass spectrometry can detect bloodborne proteins at larger scales [10], while immunohistochemistry provides spatial information about protein localization through antibody staining of tissue sections [11]. Despite advances in these experimental methods, detecting secreted proteins remains challenging due to a combination of the following reasons: the lack of detailed understanding of the protein secretion pathways in human cells and the very low concentrations of the secreted proteins in blood circulation.

To address these challenges, computational methods have been developed to predict the likelihood of proteins being secreted into the bloodstream [12]. Early computational approaches primarily leveraged machine learning with pre-constructed protein features [13–19]. These methods often relied on manually derived features from amino acid sequences, such as signal peptides (SPs) and disorder regions [20, 21], and employed downstream classifiers like Support Vector Machines (SVMs) for prediction. While these approaches offered high interpretability due to their human-engineered features, they often exhibited limited model performance and were subject to biases and uncertainties introduced by manual feature design. Recently, deep learning (DL) approaches have been proposed to achieve end-to-end feature extraction and prediction without relying on manually curated features, thereby improving the generalizability and performance of these models [22–25].

Despite the advances made using DL, these computational methods face significant challenges, including the scarcity of labeled data and the imbalance between labeled positive samples (proteins known to be secreted into blood) and a much larger set of unlabelled proteins. Additionally, many negative samples (proteins not secreted into blood) are noisy due to their derivation from incomplete information regarding protein families [13]. Although some advanced models have sought to address these issues through semi-supervised learning approaches [15, 26], they often focus on a limited protein subset, suffer from reduced interpretability due to their reliance on DL architectures, and fail to fully leverage complementary data sources.

In response to these challenges, we propose a novel interpretability-driven self-training framework named BloodProST. Our approach leverages a self-training strategy that integrates pre-constructed biologically relevant features with automatically learned features, combining labeled and pseudo-labeled data to maximize information utilization and reduce model bias. Specifically, BloodProST employs self-training, a semi-supervised learning technique that iteratively retrains the model by using its own predictions with high confidence as additional labeled data from a pool of unlabeled samples. This enables BloodProST to generalize from a limited set of labeled proteins to a broader set of unknown proteins. To mitigate noise introduced through pseudo-labeling, we incorporate domain-specific prior knowledge to constrain the distribution of predictions and enhance model performance. This is achieved through two mechanisms:

- A constraint on the proportion of predicted blood-secretory proteins (30% to 40%), based on biological evidence suggesting that $\sim $36% of human protein-coding genes are predicted to be secretory [27].
- An initial training phase using a newly constructed set of negative samples based on protein subcellular localization information from the GeneCards database [28], combined with experimentally validated positive samples.

To further enhance model interpretability and performance, BloodProST utilizes a dual-input feature extraction strategy. The model integrates pre-constructed physicochemical and sequence-derived features, such as aromaticity and molecular weight, through a CNN-based pathway for local feature extraction. Concurrently, a complementary long short-term memory (LSTM)-based pathway processes amino acid sequences to capture long-range dependencies. To identify the most informative subset of pre-constructed features and reduce computational complexity, a Differential Evolution (DE) algorithm [29] is applied for unsupervised feature selection, optimizing the silhouette score from K-Means clustering. This allows for an effective reduction in feature dimensionality before passing the data through the convolutional neural network (CNN)-based pathway.

The contributions of this work are summarized as follows:

1. We have developed BloodProST, a self-learning framework that effectively combines labeled and unlabeled data for predicting blood-secretory proteins, addressing data scarcity through a self-training strategy.
2. We have constructed a new negative protein dataset based on domain-specific knowledge using subcellular localization data from the GeneCards database, providing a balanced benchmark for model training.
3. We introduce a comprehensive set of pre-constructed physicochemical and sequence-based features, refined using DE for unsupervised feature selection to enhance interpretability and reduce computational complexity.
4. A dual-pathway architecture, combining CNN-based local feature extraction and LSTM-based sequence analysis, is proposed to capture both structural and sequential protein characteristics for robust prediction.
5. We incorporate domain-specific knowledge into the self-training process as soft or hard constraints, integrating these constraints into the training loss and pre-training BloodProST to ensure biologically meaningful predictions, thereby enhancing the model’s generalizability and reliability.

## Materials and methods

In this section, we first provide an overview of BloodProST’s key components to establish a clear conceptual framework. We then detail the construction of our benchmark dataset—alongside an independent test set to assess generalization—and describe the evaluation metrics employed. Next, we present the design of BloodProST’s inputs, including DE-based feature reduction and the dual-pathway network architecture. Finally, we outline our biologically constrained self-training strategy, which leverages both labeled and high-confidence pseudo-labeled data to improve predictive performance.

### Overview of BloodProST

**Figure 1:**
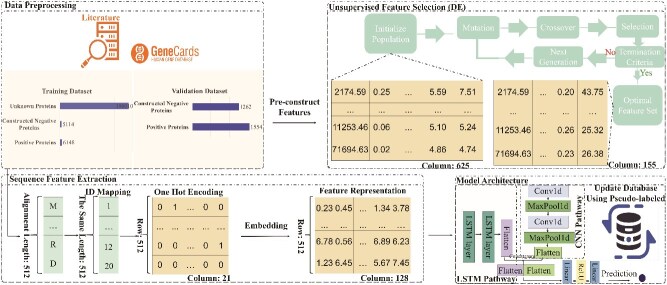
The overall workflow of BloodProST, designed for predicting which proteins can be secreted into the bloodstream.

BloodProST is a DL framework designed to predict blood-secreted proteins by integrating biologically informed feature engineering, feature selection via DE, dual-pathway feature extraction, and a self-training strategy. Figure 1 illustrates its overall workflow.

The model begins with benchmark dataset construction, comprising experimentally validated positive samples, constructed negative samples, and unlabeled proteins for self-training. From this dataset, physicochemical and sequence-based features are extracted, followed by DE-based feature selection to retain the most informative features for CNN-based extraction.

In parallel, an LSTM-based pathway processes amino acid sequences to capture long-range dependencies. The extracted features from both pathways are then concatenated into a unified representation, which serves as input for the final classification.

To overcome data scarcity, BloodProST employs a self-training strategy, iteratively generating pseudo-labels for unlabeled proteins to expand the training set. This iterative refinement significantly enhances model performance over purely supervised learning.

By integrating dataset construction, feature selection, multi-scale feature extraction, and self-training, BloodProST effectively balances interpretability, efficiency, and predictive accuracy. The following sections elaborate on these components in detail.

### Benchmark dataset

To comprehensively evaluate BloodProST, we establish benchmark datasets consisting of proteins with clearly defined secretion potentials. The datasets were divided into training, validation, and independent testing subsets, ensuring both internal validation and external generalization assessment.

#### Training and validation sets

We initially compiled two primary datasets—positive and negative samples—with distinct secretion profiles:

- **Positive samples** (*n* = 7702): These proteins were obtained from the experimentally validated Human Body Fluid Proteome dataset provided by Shao et al. [30], specifically focusing on proteins secreted into the bloodstream.
- **Negative samples** (*n* = 6376): To construct a high-confidence negative dataset representing proteins unlikely to be secreted into the bloodstream, we leveraged prior biological knowledge and subcellular localization annotations from the GeneCards database [28]. Specifically, proteins containing localization-associated keywords indicative of secretion or extracellular presence (e.g. “extracellular,” “efflux,” “synapse,” etc.; detailed fully in Supplementary Materials A) were filtered out. Additionally, we imposed a localization confidence score threshold of 1.1 to optimize the balance between dataset size and annotation accuracy, thus minimizing potential noise and ensuring dataset reliability.
- **Unknown background set** (*n* = 190 010): This set comprises all human protein entries available in the UniProt database [20] excluding those identified as positive or negative. The unknown dataset served as an unlabeled reservoir for subsequent self-training procedures.

The positive and negative sets were partitioned into training (80%) and validation (20%) subsets using a fixed random seed (42) to ensure reproducibility. All experiments involving BloodProST and baseline models were trained on the training partition and evaluated on the validation subset.

#### Independent test set

To rigorously evaluate the generalization capability of BloodProST and to minimize potential biases inherent to internal validation, we constructed an independent external test set distinct from the training and validation datasets described above:

- **Independent positive samples** (*n* = 775): These proteins were sourced from the Human Protein Atlas (HPA) Blood Protein dataset [31], which provides experimentally validated proteins explicitly confirmed to be secreted into human blood.
- **Independent negative samples** (*n* = 7769): Negative proteins were adopted from the Gold-Standard Non-Secreted Proteins (GSNP) dataset proposed by Chen et al. [32]. These samples originate from the Swiss-Prot database and were strictly defined as proteins localized exclusively to the cytoplasm or nucleus, with evidence at the protein level (Protein Existence, PE1).

Due to the larger size of the GSNP dataset relative to the HPA positive set, we implemented a robust evaluation strategy involving ten independent sampling runs. Specifically, in each run, we randomly selected a subset from GSNP negatives, equal in size to the positive set, employing unbiased random seeds ranging from 0 to 9. Subsequently, model performance metrics were computed as averages and standard deviations across these ten balanced sampling iterations.

This carefully designed benchmarking strategy ensures a comprehensive, unbiased evaluation of BloodProST and baseline models, effectively assessing both internal validity and external generalization.

### Evaluation metrics

We use six commonly employed metrics to evaluate the classification performance of BloodProST and compare it to 14 baseline models. These metrics are well-suited for imbalanced datasets and include accuracy (ACC), area under the receiver operating characteristic curve (AUC), sensitivity (SN), specificity (SP), Matthews correlation coefficient (MCC), and the F1 score.

- ACC: Represents the percentage of correctly classified instances among all instances.
- AUC: Measures the model’s ability to distinguish between positive and negative instances, providing an aggregate assessment of performance across different classification thresholds.
- SN: Also known as recall, this metric is the true positive rate, indicating the proportion of actual positives that are correctly predicted by the model.
- SP: The true negative rate, measuring the proportion of correctly predicted negatives among all actual negatives.
- MCC: A balanced metric that considers all four outcomes of a confusion matrix (true positives, true negatives, false positives, false negatives), providing a more informative score for imbalanced datasets.
- F1 score: The harmonic mean of precision and recall, balancing the trade-off between false positives and false negatives, and particularly useful for evaluating imbalanced datasets.

The mathematical definitions for these metrics are as follows:


(1) \begin{align*} & \textrm{ACC} = \frac{TP + TN}{TP + TN + FP + FN} \end{align*}

(2) \begin{align*} & \textrm{SN} = \frac{TP}{TP + FN} \end{align*}

(3) \begin{align*} & \textrm{SP} = \frac{TN}{TN + FP} \end{align*}

(4) \begin{align*} & \textrm{MCC} = \frac{TP \times TN - FP \times FN}{\sqrt{(TP + FP)(TP + FN)(TN + FP)(TN + FN)}} \end{align*}

(5) \begin{align*} & \text{F1} = \frac{2 \times \textrm{SP} \times \textrm{SN}}{\textrm{SP} + \textrm{SN}} \end{align*}

where $TP$, $TN$, $FP$, and $FN$ represent the number of true positives, true negatives, false positives, and false negatives, respectively. Higher values across these metrics indicate superior model performance, with AUC and F1 score being particularly effective for assessing imbalanced datasets [33].

### Feature construction from protein sequences

To fully leverage BloodProST’s dual-pathway architecture, we construct a comprehensive feature set that captures both physicochemical properties and sequence-based characteristics of proteins. These features complement the sequence-derived representations extracted by the LSTM pathway, enhancing model interpretability. The constructed features include molecular weight, amino acid composition AAC, secondary structure, and key physicochemical attributes, effectively representing the holistic properties of each protein. A detailed description of the computational methods used for feature extraction is provided in Supplementary Materials B.

#### Physicochemical properties

Physicochemical properties are known to play a crucial role in determining the biological functions and stability of proteins [34]. Accordingly, we compute several key physicochemical descriptors, as described below:

- Molecular weight: Calculated as the sum of the atomic weights of all amino acids in the sequence, providing a fundamental measure of protein size.
- Aromaticity: The relative frequency of aromatic amino acids (Phe, Tyr, Trp) in the sequence, which is indicative of protein stability and structural characteristics [35].
- Instability index: An empirical measure indicating the likelihood of a protein remaining stable *in vitro*; proteins with an index above 40 are considered unstable [36].
- Isoelectric point (pI): The pH at which the protein carries no net electrical charge, affecting solubility, stability, and interaction behavior [37].
- GRAVY (Grand Average of Hydropathy): A measure of overall hydrophobicity, calculated as the average hydropathy value of amino acids in the sequence.
- Net charge at pH 7: The net electrical charge of the protein at physiological pH, which plays a role in solubility and molecular interactions.
- Boman index: A measure of the protein’s binding potential, used to predict the interaction propensity with other proteins [38].
- Aliphatic index: Represents the relative volume occupied by aliphatic side chains (Ala, Val, Ile, Leu), which is associated with the thermostability of proteins [39].
- Amphipathicity: A descriptor that measures the protein’s hydrophobic and hydrophilic regions, relevant to membrane-bound and soluble proteins [40].
- Composition, transition, and distribution (CTD) descriptors: Represent a variety of physicochemical properties (e.g. polarizability, charge) as CTD indices, offering a broad view of sequence characteristics [41].

#### Sequence-derived features

To gain additional insights into the structural and functional aspects of proteins, we also compute several sequence-derived features:

- AAC: The relative frequency of each amino acid type in the protein sequence, influencing its overall function and interaction potential [42].
- Dipeptide composition: The frequencies of all possible amino acid pairs, capturing local sequence patterns and residue connectivity [43].
- Tripeptide composition: Frequencies of tripeptide combinations, providing a higher-order perspective of local sequence structure [44].
- Pseudo-AAC (PAAC): A descriptor that incorporates both sequence-order information and physicochemical properties [45].
- Amphiphilic PAAC: A variant of PAAC specifically designed to capture amphiphilic properties in protein sequences [46].
- Secondary structure fractions: Predicted proportions of helix, sheet, and random coil structures, which can significantly impact protein stability and flexibility.
- Sequence length: The total number of amino acids in a protein sequence, influencing the protein’s functional capacity and interaction dynamics.

#### Advanced features: disorder, flexibility, and aggregation

To further enhance the characterization of proteins, we included advanced descriptors related to disorder, flexibility, aggregation propensity, and cleavage sites:

- Disorder Score: A measure of the likelihood that each residue is part of a disordered region, which often affects protein flexibility and interaction potential.
- Flexibility Score: Assesses the flexibility of the protein backbone, indicative of the protein’s structural adaptiveness [47].
- Aggregation Propensity: Predicts the protein’s likelihood to aggregate by identifying aggregation-prone motifs, providing insights into potential membrane penetration or protein transport behavior.
- Cleavage Sites: The number of potential enzymatic cleavage sites within the protein sequence, offering clues to post-translational modifications PTMs and protein maturation.

Each of the features described above, ranging from sequence motifs to physicochemical properties and higher-order structural elements, provides unique insights into the protein’s structure, function, and interaction potential. This diverse feature set contributes significantly to enhancing the predictive performance of the model developed in this study, as evidenced by the ablation study on the CNN-pathway feature extraction presented in the results section.

### Differential evolution algorithm for feature selection of pre-constructed features

Feature selection is essential in bioinformatics to eliminate redundant or irrelevant features, improving model interpretability and efficiency [48]. To enhance BloodProST’s performance, we apply DE for unsupervised feature selection, optimizing a subset of pre-constructed features derived from protein sequences. The DE algorithm is guided by a k-means-based fitness function [49], identifying features that best distinguish experimentally validated positive samples from potential negative samples derived from GeneCards annotations.

#### Overview of the differential evolution algorithm

DE is a population-based stochastic optimization algorithm particularly effective for continuous optimization problems [29]. It iteratively evolves a population of candidate solutions using operations such as mutation, crossover, and selection. The main steps of DE are as follows:

1. Initialization: A population of candidate solutions is initialized randomly. Each individual in the population is represented as a continuous vector of length equal to the number of features. A vector, $\mathbf{x}_{i} = [x_{i,1}, x_{i,2}, \ldots , x_{i,n}]$, represents the selection state of each feature, where $x_{i,j} \in [0, 1]$ is a real-valued number indicating the degree of inclusion of the $j$-th feature. The initial population is generated using a uniform random distribution over the interval $[0, 1]$.
2. Mutation: For each candidate solution in the population, a mutant vector $\mathbf{v}_{i}$ is generated by adding the weighted difference between two randomly selected individuals to a third individual. This process is represented mathematically as:
  (6) \begin{align*}& \mathbf{v}_{i} = \mathbf{x}_{r1} + F \cdot (\mathbf{x}_{r2} - \mathbf{x}_{r3}),\end{align*}
  where $\mathbf{x}_{r1}, \mathbf{x}_{r2}, \mathbf{x}_{r3}$ are three distinct individuals randomly chosen from the current population, and $F$ is a scaling factor that controls the amplification of the differential variation (commonly set between 0.5 and 1 [50]). This mutation step introduces diversity into the population, enhancing exploration capabilities.
3. Crossover: A trial vector $\mathbf{u}_{i}$ is generated by performing a crossover between the mutant vector $\mathbf{v}_{i}$ and the target vector $\mathbf{x}_{i}$. The crossover operation is governed by a crossover probability $CR$, which determines how much of the mutant vector contributes to the trial vector. This is defined as:
  (7) \begin{align*}& u_{i,j} = \begin{cases} v_{i,j}, & \textrm{if } \textrm{rand }(0,1) \leq CR \textrm{ or } j = j_{\textrm{rand}}, \\ x_{i,j}, & \textrm{otherwise}, \end{cases}\end{align*}
  where $u_{i,j}$, $v_{i,j}$, and $x_{i,j}$ are the $j$-th components of the trial, mutant, and target vectors, respectively. The index $j_{\textrm{rand}}$ is randomly chosen to ensure that the trial vector differs from the target vector by at least one component.
4. Selection: The fitness of the trial vector $\mathbf{u}_{i}$ is evaluated against the target vector $\mathbf{x}_{i}$. The fitness function aims to maximize the silhouette score of clusters formed by selected features using the K-means algorithm (with $k = 2$). The silhouette score measures how similar an object is to its own cluster (cohesion) relative to other clusters (separation), and is defined as:
  (8) \begin{align*}& f(\cdot) = \frac{b - a}{\max(a, b)},\end{align*}
  where $a$ is the mean intra-cluster distance, and $b$ is the mean nearest-cluster distance for each sample. If the trial vector yields a higher silhouette score than the target vector, it replaces the target vector in the population for the next generation. The selection process is formally expressed as:
  (9) \begin{align*}& \mathbf{x}_{i}^{(g+1)} = \begin{cases} \mathbf{u}_{i}, & \textrm{if } f(\mathbf{u}_{i})> f(\mathbf{x}_{i}), \\ \mathbf{x}_{i}, & \textrm{otherwise}, \end{cases}\end{align*}
  where $f(\mathbf{u}_{i})$ and $f(\mathbf{x}_{i})$ denote the fitness values (silhouette scores) of the trial and target vectors, respectively. This iterative selection process drives the population toward optimal solutions.

By applying the DE algorithm for feature selection, we have effectively reduced the dimensionality of the pre-constructed features, lowering computational demands while enhancing model interpretability. This process prioritizes the most relevant features, enabling the CNN-based feature extraction pathway to better capture essential characteristics of proteins predictive of their secretion potential into the bloodstream. The benefits of incorporating DE are thoroughly discussed in the results section.

### Dual-pathway model structure: CNN-LSTM hybrid for protein feature extraction

BloodProST integrates CNNs [51] and LSTM networks [52] to capture both local spatial patterns and long-range sequence dependencies in protein features. The CNN pathway extracts local structural information, while the LSTM pathway models sequential relationships, enabling a complementary multi-scale feature representation that enhances classification performance.

#### Convolutional neural networks for local feature extraction

CNNs are well-suited for extracting spatial features from input data due to their hierarchical pattern recognition capabilities [53]. In BloodProST, the CNN processes pre-constructed protein features after their dimensionality has been reduced using DE. These features capture local structural and physicochemical information about the proteins and are treated as one-dimensional signals, enabling the convolutional filters to detect relevant patterns that are critical for predicting whether a protein can be secreted into the bloodstream.

The CNN pathway in BloodProST consists of two convolutional layers, each followed by ReLU activation functions and max-pooling operations (detailed formulas provided in Supplementary Materials C1):

- First Convolutional Layer: The input feature maps are processed using 64 filters of size 3, followed by a ReLU activation function [54]. This layer extracts low-level patterns from the reduced pre-constructed features, which include physicochemical and sequence-based attributes.
- Max-Pooling Operation: A max-pooling operation with a kernel size of 2 is applied to reduce the dimensionality of the feature maps, allowing the model to focus on the most significant activations and improving computational efficiency.
- Second Convolutional Layer: The output from the first convolutional layer is fed into a second convolutional layer with 128 filters, which extracts more complex patterns from the data. This is followed by another max-pooling operation, further refining the feature representation while retaining key information.

The convolution and pooling operations progressively extract local patterns and reduce the dimensionality of the feature maps, preserving the most relevant spatial characteristics. The output from the second convolutional layer is then flattened into a feature vector that represents a deeper, more abstract representation of the original protein properties. This feature vector is subsequently combined with the output from the LSTM pathway.

#### Long short-term memory networks for sequential dependencies

Operating in parallel with the CNN pathway, the LSTM pathway models long-range dependencies in protein sequences. While the CNN extracts local structural features, the LSTM captures temporal relationships, enabling the model to identify distant residue interactions that influence protein function.

The LSTM pathway follows the structure outlined below (with detailed formulas provided in Supplementary Materials C2):

- Embedding Representation: Each amino acid in the protein sequence is represented as a dense vector of fixed dimension (128 in this study). This embedding captures informative representations of amino acids based on their roles within the sequence, providing a richer input for the LSTM.
- LSTM Layer: The embedded sequence is processed by a bidirectional LSTM layer with 128 hidden units. By capturing both forward and backward dependencies, this layer provides a comprehensive understanding of the sequence. The LSTM’s gating mechanisms ensure that only the most relevant information is retained, thereby maintaining crucial long-term dependencies while discarding less important data.

#### Combined CNN-LSTM architecture

The CNN and LSTM pathways generate complementary feature representations, which are concatenated to fuse local structural patterns—extracted from pre-constructed features by the CNN—with long-range sequential dependencies learned from raw amino acid sequences by the LSTM directly along the feature dimension. This fusion enables localized interaction modeling via CNN and sequence-level context learning via LSTM, enhancing overall feature extraction.

The concatenated feature vector is subsequently passed through a fully connected layer for the final classification. By combining the CNN and LSTM, BloodProST can extract multi-scale features and leverage both structural and sequential information, leading to a more robust understanding of protein characteristics and ultimately improving the prediction of whether a protein can be secreted into the bloodstream.

### Training BloodProST with a self-training framework for pseudo-label incorporation

Self-training is an iterative semi-supervised learning approach that expands labeled datasets by generating pseudo-labels for high-confidence predictions from unlabeled data. This process refines model parameters and enhances generalization, particularly in domains with limited labeled data.

To ensure reliable pseudo-labels, BloodProST undergoes initial supervised training on experimentally validated positive samples and domain-informed negative samples. The model is then iteratively refined via incremental self-training, progressively incorporating pseudo-labeled proteins while constraining prediction distributions using biological priors. This strategy mitigates bias and guides the model toward biologically plausible predictions.

#### Overview of the self-training process

BloodProST undergoes initial supervised training using experimentally validated positive and constructed negative samples. Following this, the model iteratively predicts labels for unlabeled data. At each iteration, the top and bottom 1% of samples with the highest and lowest confidence scores are selected as pseudo-positives and pseudo-negatives, respectively, and incorporated into the training set.

This iterative self-training cycle continues until a predefined stopping criterion (e.g. maximum iterations) is met. By dynamically expanding the dataset with pseudo-labeled samples, BloodProST progressively improves generalization and exposure to diverse samples. The model’s convergence and effectiveness are evaluated in the Results section.

#### Initial supervised training with labeled data

The initial training phase employs a supervised learning approach using the labeled dataset, which consists of both positive and negative samples. To address the class imbalance between the 7702 positive and 6376 negative samples, we use a weighted binary cross-entropy loss function. The weights are inversely proportional to the class frequencies to prevent bias toward the majority class. The model is optimized using the Adam algorithm with a learning rate of $2 \times 10^{-5}$ and trained for 100 epochs. The loss function is defined as follows:


(10) \begin{align*}& \mathcal{L}_{\textrm{initial}} = - \frac{1}{N} \sum_{i=1}^{N} w_{c} \left( y_{i} \log(p_{i}) + (1 - y_{i}) \log(1 - p_{i}) \right),\end{align*}

where $w_{c}$ is the weight for class $c$ (positive or negative), $y_{i}$ is the true label of sample $i$, and $p_{i}$ is the predicted probability.

#### Self-training strategy: selection of pseudo-negatives and pseudo-positives

During each self-training iteration, BloodProST identifies high-confidence samples from the unlabeled dataset. Specifically, the top 1% of samples with the lowest predicted probabilities are assigned as pseudo-negatives, while the top 1% with the highest probabilities are assigned as pseudo-positives. This selection strategy prioritizes samples with extreme confidence scores, assuming they are more likely to be correctly classified despite some inherent noise.

Mathematically, the selection criteria are defined as follows:


(11) \begin{align*} & \text{Pseudo-negatives} = \{x \in X_{\textrm{unlabeled}}: p(x) \leq \theta_{\textrm{neg}}\}, \end{align*}

(12) \begin{align*} & \text{Pseudo-positives} = \{x \in X_{\textrm{unlabeled}}: p(x) \geq \theta_{\textrm{pos}}\}, \end{align*}

where $\theta _{\textrm{neg}}$ and $\theta _{\textrm{pos}}$ are the thresholds corresponding to the top 1% of lowest and highest predicted probabilities, respectively.

#### Incorporating biological knowledge through regularization

To align BloodProST’s predictions with biological priors, we introduce a regularization term during the self-training phase alongside the weighted binary cross-entropy loss (10). Given that $\sim $36% of human protein-coding genes are predicted to be secreted into the bloodstream [27], we incorporate a biologically informed constraint on the predicted proportion of secretory proteins. To account for potential uncertainties, we set a range of 30-40% and integrate this as a regularization constraint in the loss function. The regularization penalty is defined as follows:


(13) \begin{align*}& \mathcal{R} = \begin{cases} (\lambda_{\textrm{initial}} \cdot{\gamma}^{g}) \cdot (L - p_{\textrm{positive}}), & \textrm{if } p_{\textrm{positive}} < L, \\ (\lambda_{\textrm{initial}} \cdot{\gamma}^{g}) \cdot (p_{\textrm{positive}} - U), & \textrm{if } p_{\textrm{positive}}> U, \\ 0, & \textrm{otherwise}, \end{cases}\end{align*}

where $p_{\textrm{positive}}$ is the proportion of positive predictions (true positives and pseudo-positives combined) in the entire dataset, and $L$ and $U$ are the lower and upper bounds of the positive proportion (30% and 40%, respectively). The term $(\lambda _{\textrm{initial}} \cdot{\gamma }^{g})$ is the penalty coefficient, where $g$ is the current iteration number in self-training, and $\gamma $ is the decay factor (set to 0.95) to gradually reduce the effect of the penalty over time. The coefficient $\lambda _{\textrm{initial}}$ is set to 20 to strongly enforce this constraint early in the training process, ensuring alignment with biological knowledge before allowing the model to focus on improving accuracy in later iterations.

The overall loss function during self-training is therefore:


(14) \begin{align*}& \mathcal{L}_{\text{self-training}} = \mathcal{L}_{\textrm{initial}} + \mathcal{R},\end{align*}

where $\mathcal{L}_{\textrm{initial}}$ is the weighted binary cross-entropy loss as defined in (10).

#### Advantages of self-training in BloodProST

The self-training process iteratively refines BloodProST by retraining it on an expanded dataset, which includes both true labels and pseudo-labels. This iterative process allows the model to generalize better by exposing it to diverse pseudo-labeled samples that may change with each iteration. By incorporating pseudo-labeled data, the model benefits from an expanded training set, mitigating overfitting that could occur due to a limited number of true labeled samples. Additionally, the presence of noisy pseudo-labels provides a natural regularization effect, enhancing model robustness.

The integration of domain-specific biological knowledge through regularization further ensures that BloodProST’s predictions are biologically plausible, aligning the learned distribution with known biological constraints. By gradually reducing the penalty coefficient $\lambda $ over successive iterations, the model can focus more on improving classification accuracy after initial alignment with biological priors. The iterative refinement continues until a convergence criterion is met, such as a maximum number of iterations, leading to an optimal balance between predictive performance and biological alignment.

The impact of the self-training strategy and its effects on model performance are quantitatively assessed and discussed in detail in the Results section.

## Results

We begin by validating the reliability of our constructed negative dataset against independently curated UniProt annotations. Next, we systematically evaluate BloodProST, isolating the contributions of its three core components:

1. **DE-based feature reduction**, assessed both by quantitative class separation and by interpretability analyses;
2. **Dual-pathway CNN–LSTM architecture**, which integrates pre-constructed features and raw sequences; and
3. **Self-training strategy**, which iteratively incorporates high-confidence pseudo-labels.

We then compare BloodProST with 14 baseline models on both the internal validation set (inner-validation) and an independent test set (outer-validation). To demonstrate the robustness of our design, we perform a sensitivity analysis of the self-training regularization parameters. We further assess the biological plausibility of BloodProST’s predictions by examining secretion-related markers (e.g. SPs, transmembrane TM regions, glycosylation sites). Finally, we extend the framework to predict protein secretion into urine using the identical architecture to illustrate its generalizability.

All experiments were conducted with a fixed random seed (42) to ensure reproducibility. To emphasize model robustness, we applied consistent hyperparameter settings across all experiments without dataset-specific tuning. Detailed model configurations are provided in Supplementary Materials D.

### Validation of negative sample construction

To validate the reliability of the negative protein samples initially constructed via keyword filtering from the GeneCards database, we conduct a rigorous cross-validation using external data from the UniProt database. Specifically, we downloaded comprehensive subcellular localization annotations corresponding to our pre-constructed negative protein set from UniProt, leveraging its manually curated information on protein localization and SPs.

Each UniProt entry provides two critical annotation fields: “Subcellular location” and “SP,” both of which are fundamental determinants for evaluating whether proteins are potentially secreted into the blood. We systematically examined each protein in our constructed negative set against these annotations, applying the following exclusion criteria to confirm high-confidence negative samples:

1. The subcellular localization contains the keyword “Secreted.”
2. The subcellular localization contains the keyword “Extracellular.”
3. The SP annotation field is non-empty, indicating the presence or prediction of a SP.

**Figure 2:**
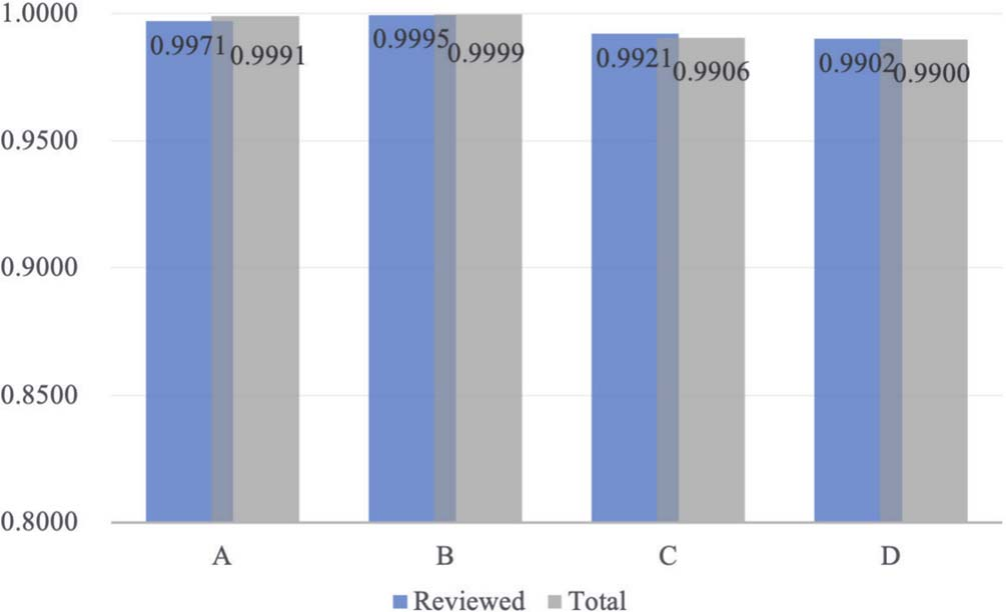
Histogram depicting the retention proportions of high-confidence negative samples after UniProt-based filtering, with bars on the left representing the Reviewed subset and bars on the right representing the Total subset across four exclusion categories.

Proteins that met none of the exclusion criteria were designated as high-confidence negatives, indicating strong evidence against their secretion potential. The proportions of retained high-confidence negatives under each filtering scenario are presented in Fig. 2. Blue bars denote the Reviewed subset (Swiss-Prot entries), valued for their expert curation, whereas gray bars denote the Total set (including both Swiss-Prot and TrEMBL entries). Categories “A” and “B” show the fractions remaining after excluding proteins annotated as ”Secreted” or ”Extracellular,” respectively; category “C” reflects exclusion based solely on the presence of a SP; and category “D” applies all three criteria concurrently, representing the most stringent validation.

Figure 2 clearly illustrates that over 99% of the pre-constructed negative samples were retained even under the strictest combined filtering conditions (“D”). Specifically, within the reviewed subset comprising 2038 original entries, 2018 (99.02%) proteins remained after applying all three exclusion criteria. Similarly, within the total subset containing 6689 original entries, 6622 (99.00%) proteins remained. This indicates that fewer than 1% of the initially GeneCards-derived negative samples could potentially be classified as secreted or extracellular based on authoritative UniProt annotations.

The exceptionally high retention rate (¿99%) obtained from this rigorous external validation effectively addresses concerns about negative-label uncertainty. By cross-verifying our initial negative set with experimentally validated and curated UniProt data, we have substantially strengthened the reliability of the negative samples used for training BloodProST. Consequently, the negative dataset is affirmed to be robust and suitable for subsequent model training and evaluation.

### Feature importance analysis

A fundamental component of BloodProST is its DE-based feature selection module. Initially comprising 625 physicochemical and sequence-derived descriptors, DE effectively reduces this set to 155 features, representing only 24.8% of the original dimensionality. Crucially, the retained features include biologically relevant attributes such as aromaticity indices, hydrophobicity scales, and proteolytic cleavage motifs, all known to play significant roles in protein secretion and processing [55, 56].

**Figure 3:**
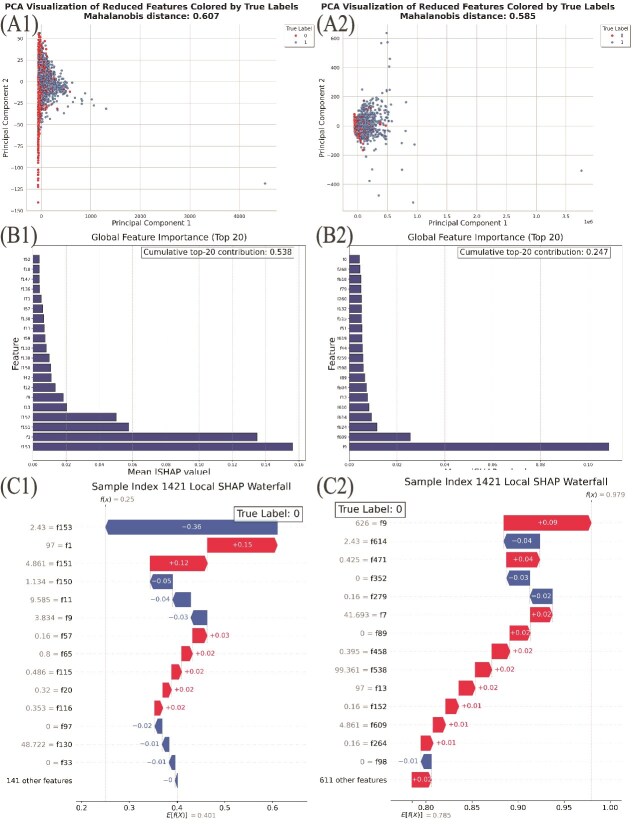
Effect of DE-based feature selection on cluster validity and model interpretability, as shown by PCA scatterplots with Mahalanobis distance indices (A1-A2), global SHAP importance for the top 20 features (B1-B2), and local SHAP waterfall plots for a representative negative-class sample (C1-C2).

To quantitatively assess the impact of DE-based reduction on feature set separability, we applied Principal Component Analysis (PCA) to both the reduced (DE-selected) and original feature sets, projecting them into two-dimensional space. Subsequently, we calculated the Mahalanobis distance, a metric quantifying the separation between different class distributions, with higher values indicating clearer distinction between clusters. As shown in Fig. 3A1 and A2, the DE-reduced feature set achieves a Mahalanobis distance of 0.607 (Fig. 3A1), compared to 0.585 for the original feature set (Fig. 3A2). These results confirm that DE-based feature selection not only reduces dimensionality but also enhances intrinsic discriminative capability between secreted (label “1”) and non-secreted (label “0”) proteins.

Beyond clustering validation, we further evaluated model interpretability through global and local SHAP analyses [57]. For this purpose, we trained two versions of the BloodProST architecture: one using the DE-reduced feature set and another using the full original feature set, computing Kernel SHAP values for both:

- **Global explanations:** Aggregating the absolute SHAP values of the top 20 features, we found a cumulative contribution of 0.538 for the DE-reduced model (Fig. 3B1), significantly higher than the cumulative contribution of 0.247 observed without DE-based reduction (Fig. 3B2). This more than two-fold increase illustrates DE’s ability to focus the model’s attention on the most predictive features.
- **Local explanations:** To provide further interpretative insights, we randomly selected a negative-class sample and visualized its SHAP waterfall plot. For the DE-reduced model (Fig. 3C1), the predicted score correctly decreased from an expected baseline value $E[f(X)] = 0.401$ to a final prediction $f(x) = 0.25$, primarily driven by feature f153 (SHAP value: $-0.36$). In contrast, the model trained on the original feature set (Fig. 3C2) produced a significantly higher final score $f(x) = 0.979$ due to predominately positive contributions, resulting in misclassification.

Taken together, the quantitative and qualitative analyses presented in Fig. 3A1–C1 clearly demonstrate that DE-based feature reduction improves the discriminative quality of the feature space and significantly enhances model interpretability by focusing predictions on a biologically meaningful and compact subset of features.

### Ablation experiments for dual-pathway design: CNN-based pathway and LSTM-based pathway

**Figure 4:**
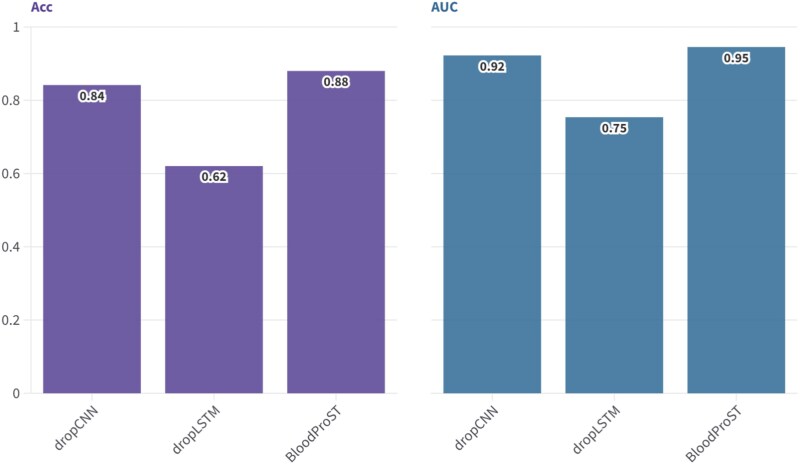
Ablation study of the dual-pathway design in BloodProST, showing ACC and AUC values for model variants omitting either the CNN-based or LSTM-based pathway.

To evaluate the contributions of each feature extraction pathway in BloodProST, we conducted an ablation study by selectively omitting either the CNN-based or the LSTM-based pathway while retaining the other. The objective was to observe the impact on model performance, specifically in terms of ACC and AUC, which collectively reflect the model’s effectiveness on the validation dataset. The results of this study are presented in Fig. 4.

Figure 4 shows that both feature extraction pathways positively contribute to BloodProST’s predictive performance for identifying blood-secreted proteins. Notably, the LSTM-based pathway plays a more critical role in the model’s performance; removing it (“dropLSTM”) results in a substantial decrease of 25.96% in ACC and 19.16% in AUC. In comparison, omitting the CNN-based pathway (“dropCNN”) leads to only a moderate decline of 3.84% in ACC and 2.29% in AUC.

The significant contribution of the LSTM-based pathway aligns well with findings from successful models like AlphaFold [58], which heavily rely on sequence information for protein structure prediction. This outcome is consistent with the fundamental principle that a protein’s amino acid sequence determines its primary structure, which serves as the foundation for its higher-order conformation [59]. The LSTM-based pathway effectively captures these sequential relationships, making it highly valuable for the prediction task.

On the other hand, the CNN-based pathway extracts features from pre-constructed feature maps, including physicochemical and sequence-derived attributes. While these features are biologically relevant, their effectiveness is inherently limited by current biological knowledge, which may not fully capture all factors influencing protein secretion.

This analysis highlights why traditional machine learning approaches, which often rely solely on manually constructed features, may struggle to achieve high performance in predicting protein secretion. These approaches are limited in their ability to capture unknown or latent features, restricting a comprehensive understanding of the biological system. The integration of the LSTM-based sequence feature extraction pathway, as implemented in BloodProST, addresses these limitations and substantially enhances predictive accuracy and robustness.

### Comprehensive analysis of the self-training strategy

To evaluate the effectiveness and rationale behind this self-training strategy, we have conducted three sets of experiments.

**Figure 5:**
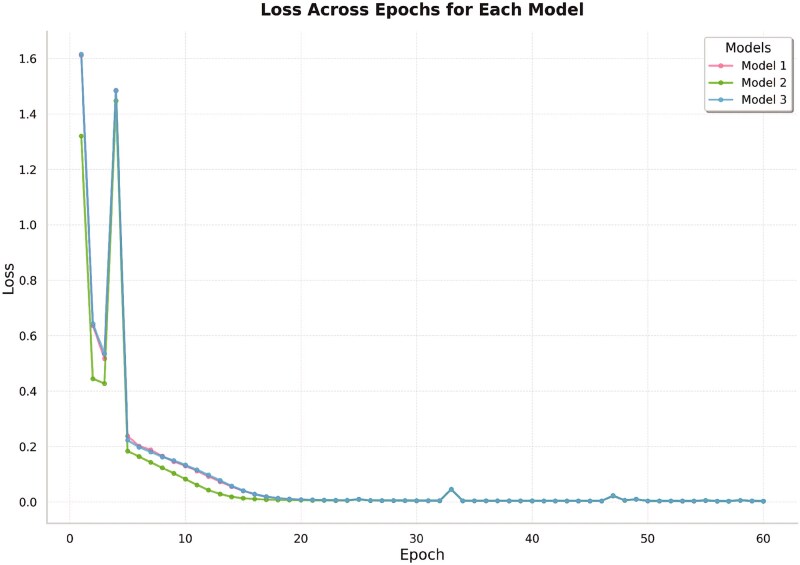
Training loss curve of three BloodProST ensemble models during self-training, plotted across training iterations.

First, we analyzed the training loss trajectory of BloodProST during self-training to assess convergence and stability, while doing the continuous inclusion of pseudo-labeled proteins. Figure 5 illustrates the loss curves for three ensemble models, each with identical architecture.

As depicted in Fig. 5, all three models demonstrate a gradual and consistent decrease in loss as the number of training iterations increases, reaching a stable convergence between iterations 40 and 60. This trend confirms that the parameters of BloodProST effectively converge despite the incorporation of pseudo-labeled samples alongside the true labeled data. Notably, we did not utilize an early stopping criterion; rather, training was conducted for a fixed maximum of 60 iterations, with the final model parameters obtained at iteration 60. Furthermore, the consistent convergence behavior observed across all ensemble models highlights the stability and robustness of our self-training strategy, suggesting that no significant noise or distributional shifts were introduced through pseudo-labeling.

**Figure 6:**
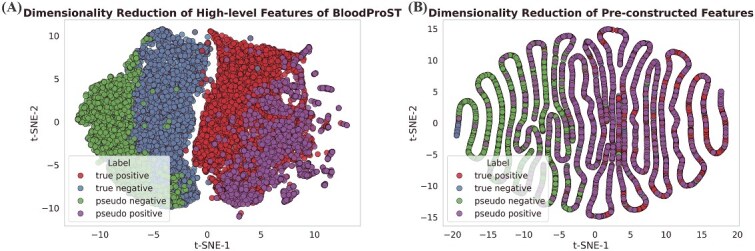
Distribution comparison between pseudo-labeled proteins generated by BloodProST and the benchmark dataset (consisting of true positive and constructed negative samples derived from GeneCards annotations), where panel (A) visualizes high-level abstract features extracted from the penultimate layer of BloodProST and panel (B) displays reduced pre-constructed features including physicochemical and sequence-based properties.

In the second experiment, we have evaluated the distributional similarity between pseudo-labeled proteins and the benchmark dataset, which comprises validated positive samples and constructed negative samples. To visualize this similarity, we employed t-distributed stochastic neighbor embedding (t-SNE) [60] to reduce the feature dimensions for visualization purposes. We considered two perspectives: one based on high-level abstract features extracted from the penultimate layer of BloodProST, and the other based on the pre-constructed features fed into the CNN pathway, including physicochemical and sequence-based properties. Figure 6 presents both visualizations, with different sample types color-coded: true positives (red), constructed negatives (blue), pseudo-positives (purple), and pseudo-negatives (green).

Figure 6A shows a clear separation between the positive and negative samples when visualizing the high-level abstract features extracted by BloodProST. Notably, pseudo-labeled samples tend to cluster closely with their corresponding true labelled counterparts, indicating the reliability of these labels. When examining the pre-constructed features, some overlap is observed between positive and negative samples, as illustrated in Fig. 6B. However, there is still a discernible trend: negative samples predominantly align along the left side of the t-SNE-1 axis, while positive samples align on the right. Quantitatively, only 1.11% of pseudo-negative samples (green) fall into the positive sample region, whereas 26.21% of pseudo-positive samples (purple) fall into the negative region. This observation, consistent with Fig. 6B, suggests that the pseudo-labeled samples maintain a distribution similar to that of their true labels. Moreover, the contrast between Fig. 6A and B underscores the advantage of DL over traditional machine learning approaches: relying solely on pre-constructed features limits the performance, while extracting deeper and more complex features enables more effective classification.

**Figure 7:**
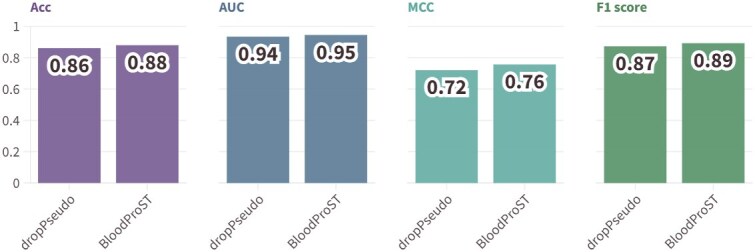
Performance comparison between BloodProST trained with and without pseudo-labeled samples across ACC, AUC, MCC, and F1 score metrics.

The third experiment assessed the impact of self-training by comparing BloodProST’s performance with and without the inclusion of pseudo-labeled samples. This comparison aimed to demonstrate the necessity and effectiveness of incorporating pseudo-labels. The results are shown in Fig. 7.

As illustrated in Fig. 7, incorporating pseudo-labeled samples significantly enhances BloodProST’s performance across multiple metrics. The original model achieves an ACC of 0.88, an AUC of 0.9454, an MCC of 0.7569, and an F1 score of 0.8926. In comparison, the model variant without pseudo-labeled samples (“dropPseudo”) experiences notable declines: ACC decreases by 1.88%, AUC by 1.04%, MCC by 3.66%, and F1 score by 1.99%. These results underscore the advantage of self-training with pseudo-labeling, which not only expands the training set but also enhances model generalizability on previously unseen data, despite the inherent noise introduced by pseudo-labels.

In summary, the self-training process incrementally refines BloodProST by retraining on an expanded dataset that includes both true and pseudo-labeled samples. The pseudo-labeled samples vary across iterations, exposing the model to a diverse set of examples and thereby improving generalization.

### Comprehensive comparisons with 14 baselines

This section presents a comparative analysis of BloodProST against 14 baseline models across six evaluation metrics. These baselines were developed specifically for this study due to the current lack of a unified benchmark dataset for predicting protein secretion into the bloodstream. To ensure fair and reliable comparisons, each baseline model was trained using the same training dataset, identical training procedures, and consistent hyperparameter settings (excluding architecture-specific parameters). All models, including BloodProST, were evaluated consistently using the same validation dataset and a rigorously curated independent test dataset. This setup ensures both fairness and comprehensiveness in the comparisons, covering traditional machine learning methods and advanced DL architectures.

The 14 baseline models are categorized according to their input data and architectural characteristics as follows:

**Models Utilizing Pre-Constructed Features:** These models rely solely on pre-constructed features, identical to those employed in BloodProST’s CNN-based feature extraction pathway. The group comprises a fully connected network (FCN-a), a CNN (CNN-a), a manifold ranking algorithm (MR-a), and three ensemble learning methods—Random Forest (RF-a), XGBoost (XGBoost-a), and SVM-a. Additionally, the sophisticated U-Net architecture [61], labeled as U-Net-a, is included to investigate if advanced architectures significantly boost performance.
**Models Using Amino Acid Sequences:** This category includes models processing protein sequences, mirroring BloodProST’s sequence-based pathway. It includes LSTM (LSTM-b), GRU (GRU-b), and Transformer (Transformer-b) [62], an advanced attention-based sequence model.
**Models Integrating Features and Sequences:** These models combine the pre-constructed features and amino acid sequences concurrently, matching BloodProST’s dual-input strategy. Architectures include LSTM-FCN-c, GRU-FCN-c, GRU-CNN-c, and Transformer-U-Net-c.

To thoroughly assess the effectiveness of the self-training strategy involving pseudo-labeled data, we evaluated 10 deep-learning-based models (excluding RF-a, XGBoost-a, SVM-a, and MR-a) under both purely supervised learning conditions and with the incorporation of self-training. Specifically, all models employed an identical self-training methodology for consistency and comparability. Performance results are summarized in Tables 1 and 2, showing performances without and with self-training, respectively.

**Table 1:** Comparison of BloodProST with 14 baselines on the validation dataset without employing self-training

Model	SN	SP	**ACC**	**MCC**	**AUC**	**F1 score**
FCN-a	0.0000	1.0000	0.4482	0.0000	0.2432	0.0000
CNN-a	0.4041	0.8788	0.6168	0.3142	0.7538	0.5379
MR-a	0.0019	1.0000	0.4492	0.0294	0.4912	0.0039
RF-a	0.7445	0.6276	0.6921	0.3747	0.6861	0.7274
XGBoost-a	0.8012	0.5753	0.6999	0.3883	0.6882	0.7466
SVM-a	0.8243	0.5222	0.6889	0.3662	0.6733	0.7452
U-Net-a	0.3777	0.8962	0.6101	0.3126	0.7539	0.5167
LSTM-b	0.8507	0.8534	0.8519	0.7021	0.9316	0.8638
GRU-b	0.8198	0.8494	0.8331	0.6662	0.9128	0.8443
transformer-b	0.7239	0.8542	0.7823	0.5764	0.8807	0.7859
LSTM-FCN-c	0.8385	0.8415	0.8398	0.6779	0.9248	0.8525
GRU-CNN-c	0.8353	0.8510	0.8423	0.6836	0.9190	0.8539
GRU-FCN-c	0.8340	0.8162	0.8260	0.6490	0.9014	0.8410
Transformer-U-Net-c	0.7233	0.8685	0.7884	0.5906	0.8890	0.7904
BloodProST (Ours)	0.8623	0.8597	**0.8612**	**0.7203**	**0.9350**	**0.8727**

Six metrics are used to assess the prediction of proteins secreted into the bloodstream.

**Table 2:** Comparison of BloodProST with 14 baselines on the validation dataset with the same self-training strategy

Model	SN	SP	**ACC**	**MCC**	**AUC**	**F1 score**
FCN-a	0.9981	0.1109	0.6005	0.2469	0.7528	0.7339
CNN-a	0.4106	0.8716	0.6172	0.3112	0.7539	0.5421
MR-a	-	-	-	-	-	-
RF-a	-	-	-	-	-	-
XGBoost-a	-	-	-	-	-	-
SVM-a	-	-	-	-	-	-
U-Net-a	0.3565	0.9176	0.6080	0.3221	0.7539	0.5009
LSTM-b	0.8578	0.8463	0.8526	0.7028	0.9323	0.8653
GRU-b	0.8288	0.8439	0.8356	0.6701	0.9122	0.8476
transformer-b	0.7272	0.8574	0.7855	0.5828	0.8784	0.7891
LSTM-FCN-c	0.8378	0.8431	0.8402	0.6787	0.9257	0.8527
GRU-CNN-c	0.8166	0.8605	0.8363	0.6737	0.9188	0.8463
GRU-FCN-c	0.8366	0.8154	0.8271	0.6510	0.9012	0.8422
Transformer-U-Net-c	0.7201	0.8700	0.7873	0.5893	0.8902	0.7889
BloodProST (Ours)	0.9041	0.8502	**0.8800**	**0.7569**	**0.9454**	**0.8926**

Six metrics are used to assess the prediction of proteins secreted into the bloodstream.

Tables 1 and 2 demonstrate that BloodProST consistently surpasses all 14 baseline models across key evaluation metrics, irrespective of the training strategy used. Particularly noteworthy is the improvement in F1 scores—a critical measure for imbalanced datasets—observed in 72.73% of DL architectures upon the inclusion of pseudo-labeled data via self-training. This outcome aligns with prior literature suggesting that pseudo-labeling can often outperform purely human-annotated datasets [63, 64].

Moreover, models leveraging sequence-based inputs or a combination of sequences and pre-constructed features (categories “b” and “c”) consistently outperformed models restricted to pre-constructed features alone (category “a”). This aligns with BloodProST’s ablation results, where omitting the LSTM-based sequence extraction pathway caused substantial performance declines (25.96% reduction in ACC and 19.16% reduction in AUC) compared to omitting the CNN-based pathway. This underscores the intrinsic value of sequence-derived features, capturing critical structural and functional information inadequately represented by manually engineered features alone.

Interestingly, despite its sophisticated attention mechanism, the Transformer-based model exhibited lower performance compared to simpler LSTM and GRU architectures in multiple metrics (ACC, AUC, MCC, and F1 score). This likely reflects underfitting due to the limited dataset size, highlighting the necessity of aligning model complexity with available data.

Next, to further evaluate the robustness and generalization capabilities of BloodProST beyond internal validation, we conducted additional independent tests using a fully external dataset (HPA-GSNP test set). The independent evaluation results, presented in Table 3, summarize mean and standard deviation performance metrics across ten balanced sampling runs. BloodProST achieved the highest mean AUC (0.8543 $\pm $ 0.0337), notably outperforming all baseline models. Additionally, its Matthews correlation coefficient (MCC) of 0.4044 ($\pm $ 0.0402) and F1 score of 0.7361 ($\pm $ 0.0130) indicate balanced classification capabilities. Although BloodProST’s F1 score slightly trails behind architectures such as LSTM-b, LSTM-FCN-c, and GRU-CNN-c, it exhibits lower variability across repeated independent tests (lowest standard deviation among models), reflecting stable and consistent performance across varied testing conditions. Such stability is crucial for practical applications in large-scale proteomic analyses.

**Table 3:** Performance comparison of various model architectures on the independent HPA–GSNP test set

Model	SN	SP	**ACC**	**MCC**	**AUC**	**F1 score**
FCN-a	0.9821 (0.0000)	0.0054 (0.0114)	0.4938 (0.0057)	-0.0704 (0.0506)	0.2190 (0.0292)	0.6599 (0.0026)
CNN-a	0.0536 (0.0000)	0.6946 (0.0418)	0.3741 (0.0209)	-0.3271 (0.0396)	0.2194 (0.0289)	0.0789 (0.0024)
U-Net-a	0.0536 (0.0000)	0.7589 (0.0312)	0.4062 (0.0156)	-0.2636 (0.0326)	0.2194 (0.0290)	0.0828 (0.0020)
LSTM-b	0.9643 (0.0000)	0.3661 (0.0467)	0.6652 (0.0234)	0.4116 (0.0404)	0.8448 (0.0231)	0.7425 (0.0134)
GRU-b	0.9286 (0.0000)	0.3554 (0.0482)	0.6420 (0.0241)	0.3455 (0.0450)	0.8249 (0.0234)	0.7220 (0.0135)
Transformer-b	0.6321 (0.0279)	0.5643 (0.0577)	0.5982 (0.0314)	0.1971 (0.0626)	0.6180 (0.0378)	0.6116 (0.0249)
LSTM-FCN-c	0.9464 (0.0000)	0.3857 (0.0630)	0.6661 (0.0315)	0.3999 (0.0558)	0.8500 (0.0233)	0.7396 (0.0182)
GRU-CNN-c	0.9464 (0.0000)	0.4125 (0.0721)	0.6795 (0.0360)	0.4231 (0.0633)	0.8157 (0.0261)	0.7476 (0.0210)
GRU-FCN-c	0.8929 (0.0000)	0.3554 (0.0403)	0.6241 (0.0201)	0.2936 (0.0394)	0.7522 (0.0252)	0.7039 (0.0111)
Transformer-U-Net-c	0.4750 (0.0368)	0.6643 (0.0413)	0.5696 (0.0359)	0.1420 (0.0735)	0.6052 (0.0402)	0.5246 (0.0393)
BloodProST (Ours)	1.0000 (0.0000)	0.2821 (0.0478)	0.6411 (0.0239)	0.4044 (0.0402)	0.8543 (0.0337)	0.7361 (0.0130)

All models were trained using an identical self-training strategy on the same training dataset. Metrics are reported as mean ($\pm $ standard deviation) over ten balanced sampling runs.

In conclusion, these comprehensive evaluations confirm that BloodProST provides robust, reliable, and generalizable predictions, effectively prioritizing true bloodstream-secreted proteins and demonstrating substantial practical utility for real-world protein screening tasks.

### Sensitivity analysis of regularization weight settings

Building upon the self-training strategy analyzed above, we further investigated the sensitivity of BloodProST to variations in its regularization hyperparameters. Specifically, BloodProST employs a dynamically adjusted regularization term defined by a prior weight and a decay factor, controlling the distribution of prediction during iterative training. Our default parameter setting in BloodProST utilizes an initial prior weight of 20 and a decay factor of 0.95. The initial prior weight of 20 was chosen to enforce a roughly five-fold stronger constraint relative to the first loss term at the early stages of training, thereby aligning predictions closely with established biological priors.

To evaluate sensitivity to these parameters, we compared the default BloodProST configuration against four variant models:

- **Variant 1 (weaker prior)**: prior weight = 6, decay = 0.95.
- **Variant 2 (stronger prior)**: prior weight = 60, decay = 0.95.
- **Variant 3 (no decay)**: prior weight = 20, decay = 1.00.
- **Variant 4 (rapid decay)**: prior weight = 20, decay = 0.90.

**Figure 8:**
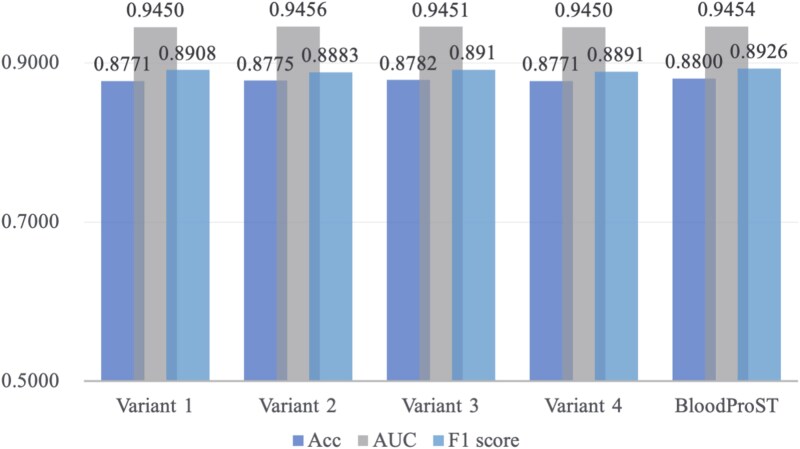
Validation performance of BloodProST and its four regularization variants, measured by ACC, AUC, and F1 score.

Figure 8 summarizes the validation performance of each variant in terms of Acc (dark blue), AUC (gray), and F1 score (light blue). Across these variants, the observed performance metrics exhibited only minimal fluctuations relative to the default BloodProST model. Specifically, variations in performance were consistently minor, indicating a robust stability of the model against moderate adjustments in both the prior weight and the decay factor. This robustness demonstrates that the self-training strategy employed by BloodProST does not critically depend on finely tuned hyperparameters. Furthermore, the minimal variation in performance underscores the reliability of the pseudo-labeled data used during iterative training. Therefore, BloodProST can reliably achieve high-quality predictive performance without requiring extensive hyperparameter optimization for the regularization term.

### Reliability verification of model predictions: signal peptides, transmembrane regions, and additional secretion markers

Following the comparison of BloodProST with 14 baseline models, demonstrating its superior performance, we further assess the reliability and biological plausibility of the pseudo-labeled samples generated by BloodProST. In this validation, we focused on multiple secretion-related motifs and PTMs known to correlate with protein secretion or cell-surface localization. Specifically, we extract and evaluate the following biologically relevant features from UniProt annotations for both pseudo-positive and pseudo-negative samples:

1. **SP**: Presence of an N-terminal signal sequence.
2. **TM region**: One or more $\alpha $-helical membrane spans.
3. **N-linked glycosylation**: Consensus N-glycosylation sites.
4. **Disulfide bond (SS)**: Annotated cysteine pairs forming disulfide bridges.
5. **Glycosylphosphatidylinositol-anchor (GPI)**: GPI attachment sites.
6. **ER-retention motif**: C-terminal sequences (KDEL (Lys-Asp-Glu-Leu), HDEL (His-Asp-Glu-Leu), KEEL (Lys-Glu-Glu-Leu), QEEL (Gln-Glu-Glu-Leu)), typically indicative of endoplasmic reticulum residency (calculated from sequences).
7. **Dibasic cleavage site**: Presence of “KR” or “RR” motifs within the last 20 residues, often guiding proteins into secretory pathways (calculated from sequences).

**Figure 9:**
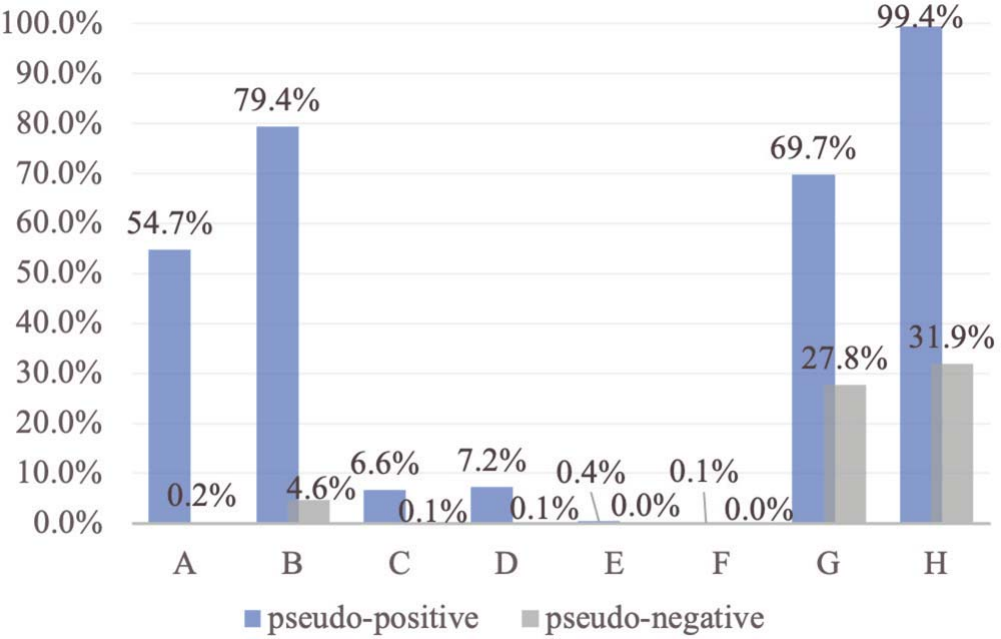
Proportion of pseudo-positive (bars on the left) and pseudo-negative (bars on the right) samples exhibiting various secretion-related markers: (A) SP, (B) TM region, (C) N-glycosylation, (D) SS, (E) GPI-anchor, (F) ER-retention motif, (G) Dibasic cleavage site, and (H) at least one of the above markers (overall indicator).

The proportion of pseudo-positive and pseudo-negative proteins containing these features was systematically quantified. Additionally, we calculated an overall indicator, defined as the presence of at least one of these secretion-related markers. The results are summarized in Fig. 9, with labels A–H corresponding to the respective markers.

From Fig. 9, we observe that BloodProST’s pseudo-positive samples exhibit significantly higher frequencies of all evaluated markers compared to the pseudo-negative samples. This pronounced difference aligns well with established biological knowledge, thereby strongly supporting the biological validity of the pseudo-labels. Most notably, 99.4% of the pseudo-positive proteins contain at least one secretion-related marker, whereas only 31.9% of pseudo-negative proteins display any marker. This substantial contrast reinforces the reliability of BloodProST’s pseudo-positive predictions, demonstrating consistency with known biological features of secreted and membrane-associated proteins.

These findings underscore BloodProST’s capability to effectively capture diverse secretion signals beyond the classical indicators such as SPs and TM regions, despite these specific features not being explicitly included in the initial pre-constructed feature set. The high prevalence of secretion markers among pseudo-positive samples and their minimal occurrence among pseudo-negative samples affirm that our self-training strategy generates biologically meaningful pseudo-labels. Consequently, this comprehensive, marker-based validation substantially alleviates concerns regarding the accuracy and biological relevance of the pseudo-labeled samples, confirming BloodProST’s robustness in predicting blood-secreted proteins.

### Clinical perspective and extension to urine proteome prediction

Early cancer detection markedly improves patient outcomes, as most tumors are curable at an incipient stage [65]. Owing to its rich molecular diversity, the blood proteome represents a premier source for biomarker discovery [66]. Leveraging RNA-seq data from healthy individuals Genotype-TissueExpression (GTEx), cancer patients The Cancer Genome Atlas (TCGA), and non-cancer controls Gene Expression Omnibus (GEO), we used a random-forest classifier to nominate eight tumor-specific, up-regulated blood proteins as putative pan-cancer biomarkers. Plasma samples from healthy donors and patients with several cancer types have been collected, and both Astral-DIA proteomics and ELISA assays are underway to validate the diagnostic utility of these candidates (in preparation).

To further demonstrate the generalizability of the BloodProST architecture beyond blood-secreted proteins, we evaluated its performance in predicting protein secretion into urine. Positive samples ($n=3{}880$) were extracted from the Human Urine PeptideAtlas [67], while negative samples ($n=6376$) comprised high-confidence non-secreted proteins previously curated from GeneCards (as detailed in Methods). We combined these datasets and performed a randomized 80%/20% split, yielding training ($n=8204$) and validation sets ($n=2052$). Subsequently, BloodProST and the ten baseline deep-learning architectures were trained using identical data splits, the same self-training strategy, and consistent hyperparameters except those unique to each architecture’s design. The resulting validation performance, measured across six evaluation metrics, is summarized in Table 4.

**Table 4:** Validation performance of BloodProST and baseline models on the urine proteome secretion prediction task

Model	SN	SP	**ACC**	**MCC**	**AUC**	**F1 score**
FCN-a	0.0000	1.0000	0.6155	0.0000	0.2433	0.0000
CNN-a	0.0000	0.9865	0.6072	−0.0722	0.5518	0.0000
U-Net-a	0.2345	0.9588	0.6803	0.2942	0.7544	0.3606
LSTM-b	0.7934	0.9351	0.8806	0.7455	0.9504	0.8363
GRU-b	0.7605	0.9240	0.8611	0.7033	0.9331	0.8081
Transformer-b	0.6324	0.9390	0.8212	0.6187	0.9043	0.7311
LSTM-FCN-c	0.7820	0.9256	0.8704	0.7234	0.9409	0.8227
GRU-CNN-c	0.7921	0.9089	0.8640	0.7103	0.9317	0.8175
GRU-FCN-c	0.7731	0.9002	0.8514	0.6830	0.9156	0.8000
Transformer-U-Net-c	0.7123	0.9089	0.8333	0.6428	0.9095	0.7667
**BloodProST (Ours)**	0.8378	0.9264	**0.8923**	**0.7711**	**0.9618**	**0.8568**

BloodProST achieved the best overall performance across all evaluation metrics, notably yielding an AUC of 0.9618 and an F1 score of 0.8568—two critical indicators for assessing predictive capability on imbalanced datasets. These results markedly outperform all competing models—whether based solely on pre-constructed features, solely on sequence information, or on their combination. Collectively, these findings validate that BloodProST’s architecture and training framework generalize effectively to other biofluids, underscoring its potential as a broadly applicable secretion prediction platform. Future work will involve the collection and integration of additional annotated datasets from diverse biofluids (e.g. saliva, cerebrospinal fluid) to further demonstrate BloodProST’s versatility.

## Conclusion

In this study, we introduced BloodProST, a novel self-learning framework for predicting blood-secreted proteins. By effectively integrating labeled and unlabeled data via a self-training strategy, BloodProST overcomes the scarcity of annotated secretion data and yields robust, interpretable predictions. Its dual-pathway architecture combines pre-constructed physicochemical features with sequence-derived embeddings, capturing both local and long-range dependencies in protein sequences.

Moreover, our DE—based feature selection module enhances interpretability and computational efficiency by isolating the most informative subset of features. When benchmarked against 14 state-of-the-art models, BloodProST consistently achieved superior performance across multiple metrics, demonstrating the added value of pseudo-labeled data and domain-specific constraints in improving model accuracy and generalizability. The alignment of BloodProST’s predictions with known biological markers—such as SPs and TM regions—further validates its reliability.

Looking forward, we plan to extend BloodProST to distinguish among different secretion pathways and functional classes (e.g. hormones, cytokines, enzymes, and immunoglobulins). Such an extension will require:

- Curating pathway-specific gold-standard labels (e.g. classical ER/Golgi versus non-classical secretion; antibody versus hormone secretion);
- Adapting the self-training loss to incorporate pathway-specific priors.

As more high-quality, functionally annotated secretion data become available—potentially from targeted proteomic studies—BloodProST can be generalized into a multi-class secretion-pathway predictor.

**Key Points**

- **Innovative Self-Training Strategy:** BloodProST employs a self-training framework that iteratively augments the limited set of experimentally validated (labeled) proteins with high-confidence pseudo-labeled samples from a large pool of unlabeled data, effectively addressing data scarcity in blood-secretory protein prediction.
- **Dual-Pathway Architecture:** The framework integrates a CNN-based pathway that processes pre-constructed physicochemical and sequence-derived features and an LSTM-based pathway that captures long-range dependencies from raw protein sequences, thereby providing a complementary multi-scale feature extraction mechanism.
- **Unsupervised Feature Selection:** DE is used to optimize feature selection by maximizing the Silhouette Score from K-Means clustering, which reduces dimensionality and improves model interpretability without sacrificing essential biological information.
- **Integration of Domain-Specific Knowledge:** Biological priors—such as the expected proportion of blood-secretory proteins (30%-40%) and subcellular localization data from GeneCards—are embedded into the training loss and pre-training process, ensuring that model predictions remain biologically plausible.
- **Superior Performance:** Comprehensive experiments demonstrate that BloodProST consistently outperforms 14 state-of-the-art models across multiple evaluation metrics (including ACC, AUC, MCC, and F1 score), achieving robust and reliable predictions that align with known biological insights on SPs and TM regions.

## Supplementary Material

Supplementary_Materials_bbaf385

## Data Availability

The code and all datasets (training, validation, independent test) are openly accessible at https://github.com/Mxc666/BloodProST.git.
