# Supplementary material for: BloodProST: prediction of blood-secretory proteins through self-training
Source: Brief Bioinform. 2025 Aug 1;26(4):bbaf385. doi: 10.1093/bib/bbaf385 (PMC12315548; doi:10.1093/bib/bbaf385)
Supplement: Supplementary_Materials_bbaf385 [file supplementary_materials_bbaf385.pdf]

Supplementary Materials

A. Keywords for Filtering Subcellular Locations from GeneCards Predictions

The following keywords are used to filter out proteins from the potential negative dataset based on subcellular location descriptions from GeneCards. These terms represent cellular components or functions that indicate proteins are not likely to be secreted into the bloodstream, thus reducing noise in the negative dataset:

- Keywords: ['golgi', 'plasma membrane', 'extracellular', 'apical', 'anchoring', 'junction', 'microtubule', 'synapse', 'efflux', 'axon', 'basement membrane', 'cargo', 'projection', 'pole', 'surface', 'bud', 'ciliary', 'collagen', 'cytoskeleton', 'dendritic', 'channel', 'katanin', 'outer', 'photoreceptor', 'synap', 'secretory', 'cell wall', 'export', 'neuronal cell body membrane', 'receptor']
- Prefixes to Exclude: ['exo', 'extra', 'kera']

These filters help in refining the selection of potential negative proteins to ensure the dataset contains minimal noise.

B. Detailed Methodology for Pre-Constructed Physicochemical and Sequence-Based Features

To comprehensively understand the structural and functional properties of proteins, we employ various computational methods to derive several physicochemical and sequence-based features. In addition to some features calculated in-house, using custom-designed formulas, we utilized Python libraries such as `BioPython` and `PyPro`, which are widely used in computational biology for sequence analysis.

1. Physicochemical Properties

- **Molecular Weight**: Represents the sum of the atomic weights of all the amino acids in the protein sequence, providing an estimate of the protein's size and mass. The calculation follows:

$$\text{Molecular Weight} = \sum_{\text{AA}} (\text{atomic weight of AA})$$

- **Aromaticity**: Defined as the relative frequency of aromatic amino acids (Phe, Tyr, Trp) within the protein sequence. It is computed as follows:

$$\text{Aromaticity} = \frac{\text{Number of aromatic residues}}{\text{Total number of residues}}$$

- **Instability Index**: Predicts protein stability based on its sequence. Proteins with an instability index greater than 40 are considered unstable. It is computed as:

$$\text{Instability Index} = 10 \times \left(\frac{\text{Sum of instability weights for all pairs}}{\text{Number of pairs}} \right)$$

- **Isoelectric Point (pI)**: The pI represents the pH at which a protein carries no net charge. It is computed by iteratively adjusting the pH and calculating the net charge of the protein until it reaches zero.
- **GRAVY (Grand Average of Hydropathy)**: The GRAVY score represents the sum of hydropathy values of all amino acids, divided by the length of the sequence, providing an estimate of the protein's overall hydrophobicity:

$$\text{GRAVY} = \frac{\sum_{\text{AA}} (\text{hydropathy of AA})}{\text{Length of sequence}}$$

- **Charge at pH 7**: Represents the net charge of the protein at a neutral pH (7.0), calculated using the `charge_at_pH()` function from `BioPython`. The charge is determined based on the pKa values of ionizable groups in amino acid residues.
- **Boman Index**: Predicts the binding potential of a protein to other proteins or molecules. It is calculated by summing the potential binding energies of all residues and normalizing by the sequence length:

$$\text{Boman Index} = \frac{\sum_{\text{AA}} (\text{binding energy of AA})}{\text{Length of sequence}}$$

- **Aliphatic Index**: Measures the relative volume occupied by aliphatic side chains (Ala, Val, Ile, Leu), which is associated with protein thermal stability. It is computed as follows:

$$\text{Aliphatic Index} = \frac{\sum_{\text{Aliphatic Side Chains}} (\text{Relative Contribution})}{\text{Length of sequence}}$$

The contributions for Ala, Val, Ile, and Leu are assigned as 1, 3, 3, and 3, respectively.

- **Amphipathicity**: Measures the balance of hydrophobic and hydrophilic regions in a protein sequence. It is computed using the hydrophobic moment method:

$$\text{Amphipathicity} = \frac{\sum_{\text{AA}} (\text{hydropathy scale value of AA})}{\text{Length of sequence}}$$

2. Sequence-Derived Features

- **Amino Acid Composition (AAC)**: Represents the frequency of each amino acid type in the sequence, offering insights into the overall amino acid composition, which influences protein structure and function.
- **Dipeptide Composition (DPC)**: Represents the frequency of all possible dipeptide pairs (two consecutive amino acids) in the sequence, capturing local sequence environments and amino acid connectivity.
- **Tripeptide Composition (TPC)**: Similar to DPC, TPC measures the frequencies of tripeptide combinations, providing a more comprehensive representation of local sequence motifs.
- **Composition, Transition, and Distribution (CTD) Descriptors**: Capture three distinct sequence properties: composition (frequency of amino acid groups with specific physicochemical properties), transition (frequency of transitions between different groups), and distribution (relative position of residues along the sequence). These are computed using the `GetCTD()` function from `PyPro`.
- **Pseudo-Amino Acid Composition (PAAC)**: PAAC incorporates sequence-order information alongside physicochemical properties, capturing both primary sequence and structural attributes. Computed using the `GetPAAC()` function from `PyPro`.
- **Amphiphilic Pseudo-Amino Acid Composition (APAAC)**: APAAC is an extension of PAAC that specifically captures amphiphilic properties, providing insights into the balance between hydrophobic and hydrophilic regions of the sequence. Computed using the `GetAPAAC()` function from `PyPro`.
- **Secondary Structure Fractions**: Represents the predicted proportions of alpha-helices, beta-sheets, and random coils in the protein. These fractions are predicted using the Chou-Fasman algorithm integrated into `BioPython`.
- **Length**: Represents the length of the protein sequence, a fundamental property that affects the protein's functionality and interaction profile.

3. Advanced Features Based on Disorder, Flexibility, and Aggregation

- **Disorder Score**: Calculated based on the amino acid composition, representing the proportion of residues that contribute to disorder, divided by the length of the protein.
- **Flexibility Score**: Derived from the disorder score, the flexibility score is calculated as:

$$\text{Flexibility Score} = 1 - \text{Disorder Score}$$

  This score reflects the overall flexibility of the protein backbone and its potential for conformational changes.
- **Aggregation Score**: Predicts the propensity of the protein to form aggregates. The score is determined by identifying aggregation-prone motifs (e.g., "VQIVYK", "NFGAIL", "VVVVV", "YYYTR") in the sequence and normalizing their frequency by the sequence length.
- **Cleavage Sites**: Represents the number of potential cleavage sites within the protein sequence, based on the presence of cleavage-prone residues (e.g., lysine and arginine). This provides insights into possible post-translational modifications.

C. Detailed Descriptions and Mathematical Formulations for CNN and LSTM Networks

This section provides a detailed explanation of the Convolutional Neural Network (CNN) and Long Short-Term Memory (LSTM) networks used in the hybrid model architecture of BloodProST. We focus on the mathematical operations involved in these networks, offering a comprehensive understanding of their mechanisms and their roles in feature extraction from physicochemical properties and protein sequences.

1. Convolutional Neural Network (CNN) for Local Feature Extraction

CNNs are a type of deep neural network particularly suited for spatial data processing. In our model, CNNs are used to extract features from the pre-constructed properties of proteins, which include physicochemical properties and some sequence-based features. The primary operations in CNN include convolution and pooling.

In the convolution operation, which is fundamental to CNNs, given an input feature map $\mathbf{X} \in \mathbb{R}^{H\times W\times C}$, where H is the height, W is the width, and C is the number of input channels, the convolutional layer applies a set of filters $\mathbf{K} \in \mathbb{R}^{F\times F\times C}$ to extract local patterns. The output of a convolutional operation is given by:

$$\mathbf{Y}_{ijc} = \sum_{c'=1}^{C}\sum_{m=1}^{F}\sum_{n=1}^{F}\mathbf{K}_{mn}^{c'} \cdot \mathbf{X}_{(i+m)(j+n)c'}$$

where $\mathbf{Y}$ is the output feature map, and i and j are the spatial coordinates in the output. The indices c, c', m, and n represent the output channel, input channel, and spatial dimensions of the filter, respectively.

The convolution operation slides the filter over the input feature map, computing a weighted sum at each position. To retain the original spatial dimensions of the input, we set the stride equal to 1 in this study. Additionally, we employ the Rectified Linear Unit (ReLU) activation function in an element-wise manner:

$$\text{ReLU}(x) = \max(0, x)$$

This function introduces non-linearity into the network, enabling the model to learn complex representations.

Pooling reduces the spatial dimensions of the output feature maps $\mathbf{Y}$, preserving essential information while improving computational efficiency. In our model, max pooling is used:

$$\mathbf{Y'}_{ij} = \max(\mathbf{Y}_{(i+k)(j+k)})$$

where k is the pooling kernel size. A max-pooling operation with a kernel size of 2 is applied to reduce the dimensionality by half in both the width and height of the feature maps, focusing on the most salient features in local regions.

2. Long Short-Term Memory Networks (LSTM) for Sequential Dependencies

LSTM networks are specifically designed to capture temporal dependencies in sequential data. To efficiently extract temporal dependencies from protein sequences, we first embed each amino acid into dense vectors of fixed dimensions, and then use the LSTM to capture long-range dependencies within the embedded amino acid sequences, such as interactions between residues that are far apart in the sequence but critical to protein structure and function.

- **Embedding Representation**

  Before feeding the amino acid sequences into the LSTM, we convert the amino acid symbols into dense vectors of fixed dimensions using an embedding representation. This embedding captures the semantic properties of the amino acids and provides a more informative representation for the LSTM.

  Given an input protein sequence $\mathbf{x} = [x_1, x_2, \ldots, x_n]$, we take the i-th amino acid x_i as an example. We first convert it to a one-hot encoded vector: $\mathbf{x}_{\text{oh}} = [0, \ldots, 0, \underbrace{1}_{i\text{-th}}, 0, \ldots, 0] \in \mathbb{R}^N$. The length of the one-hot vector (N) depends on the total number of amino acid types considered, which is 21 in this study (including 20 common amino acids and 1 special character for unknown amino acids).

  The dense representation of the i-th amino acid x_i is then computed using the embedding matrix:

$$\mathbf{e}_x^i = \mathbf{x}_{\text{oh}}^\top E = \mathbf{x}_{\text{oh}}^\top \begin{pmatrix} \mathbf{e}_0 \\ \vdots \\ \mathbf{e}_{20} \end{pmatrix}$$

  Where E is the embedding matrix, containing 21 rows where each row corresponds to an amino acid. The number of columns in the matrix defines the embedding dimension (128 in this study). The embedding weights are initialized randomly and updated during training.
- **LSTM Cell Operations**

  After obtaining the dense representation of each amino acid, we use an LSTM to extract temporal dependencies from the encoded amino acid sequence. Each LSTM cell maintains a cell state $\mathbf{C}_t$ and a hidden state $\mathbf{h}_t$, which are updated through a series of gating mechanisms. The primary operations within an LSTM cell at time step t are as follows:

Input Gate:

The input gate determines how much new information should be retained at the current time step t. It consists of the update gate $\mathbf{i}_t$ and the candidate cell state $\tilde{\mathbf{C}}_t$:

$$\mathbf{i}_t = \sigma(\mathbf{W}_i \mathbf{x}_t + \mathbf{U}_i \mathbf{h}_{t-1} + \mathbf{b}_i)$$
$$\tilde{\mathbf{C}}_t = \tanh(\mathbf{W}_C \mathbf{x}_t + \mathbf{U}_C \mathbf{h}_{t-1} + \mathbf{b}_C)$$

where $\mathbf{x}_t$ is the input at time t, $\mathbf{h}_{t-1}$ is the previous hidden state, and $\mathbf{W}_i$, $\mathbf{U}_i$, $\mathbf{W}_C$, $\mathbf{U}_C$, $\mathbf{b}_i$, and $\mathbf{b}_C$ are learnable parameters of the LSTM. σ and tanh are the sigmoid and hyperbolic tangent activation functions, respectively.

Forget Gate:

The forget gate determines which information from the previous cell state $\mathbf{C}_{t-1}$ should be discarded:

$$\mathbf{f}_t = \sigma(\mathbf{W}_f \mathbf{x}_t + \mathbf{U}_f \mathbf{h}_{t-1} + \mathbf{b}_f)$$

Cell State Update:

The cell state is updated by combining the old state (controlled by the forget gate) and the new candidate cell state (controlled by the input gate):

$$\mathbf{C}_t = \mathbf{f}_t \odot \mathbf{C}_{t-1} + \mathbf{i}_t \odot \tilde{\mathbf{C}}_t$$

where $\odot$ denotes element-wise multiplication.

Output Gate:

The output gate controls the hidden state $\mathbf{h}_t$, which serves as the output of the LSTM cell and as input to the next time step:

$$\mathbf{o}_t = \sigma(\mathbf{W}_o \mathbf{x}_t + \mathbf{U}_o \mathbf{h}_{t-1} + \mathbf{b}_o)$$
$$\mathbf{h}_t = \mathbf{o}_t \odot \tanh(\mathbf{C}_t)$$

The final output of the LSTM is a sequence of hidden states $\mathbf{h}_t$, which is flattened into a fixed-size vector representing long-range dependencies and interactions in the sequence.

- **Bidirectional LSTM Employment**

  In this study, we employ a bidirectional LSTM to extract long-distance temporal dependencies. This structure comprises two separate LSTMs that process the sequence in both forward and backward directions, enabling the model to capture dependencies from both past and future contexts, thereby providing a more comprehensive understanding of the sequence.

3. Integration of LSTM and CNN Outputs

The outputs from the CNN-based and LSTM-based pathways are concatenated to form a unified feature vector that captures both local patterns from pre-constructed features (physicochemical and sequence-based) and long-range dependencies from sequence-derived features. This combined vector is then passed through a fully connected layer for final classification. The overall architecture leverages multi-scale feature extraction, providing an enriched representation of the protein data.

Table 1. Detailed parameters used in BloodProST

Parameter Module	Name	Value
Training Phase	Batch Size	128
	Initial Epochs for Pre-training	100
	Epochs for Self-training	10
	Iterations of Self-training	60
CNN-based	Kernel Size	3
	Padding Size	1
	Max Pooling Size	2
	Output Size of First Convolution	64
	Output Size of Second Convolution	128
LSTM-based	Embedding Dimension	128
	Maximum Sequence Length	512
	Number of Layers	2
DE Parameters	Population Size	100
	Maximum Iterations	50
	Crossover Probability	0.7
	Mutation Probability	0.2

D. Detailed Parameters of BloodProST

This section provides detailed information about the parameters employed in BloodProST, focusing on three key modules: the training phase, hyperparameters of the BloodProST model, and the parameters used in Differential Evolution (DE) for dimensionality reduction of pre-constructed features.

Unless noted otherwise, every experiment was initialised with the same random seed (42) to guarantee reproducibility. All models were trained and evaluated on an NVIDIA A800 80 GB GPU; BloodProST and the baseline methods required approximately 12 hours to complete a full self-training cycle under these settings.
